# Towards the Exploitation of Physical Compliance in Segmented and Electrically Actuated Robotic Legs: A Review Focused on Elastic Mechanisms

**DOI:** 10.3390/s19245351

**Published:** 2019-12-04

**Authors:** Jie Chen, Zhongchao Liang, Yanhe Zhu, Chong Liu, Lei Zhang, Lina Hao, Jie Zhao

**Affiliations:** 1 School of Mechanical Engineering and Automation, Northeastern University, Shenyang 110819, China; chenjie@me.neu.edu.cn (J.C.);; 2 State Key Laboratory of Robotics and System, Harbin Institute of Technology, Harbin 150001, China

**Keywords:** physical compliance, robotic legs, electrically actuated, series compliance, parallel compliance

## Abstract

Physical compliance has been increasingly used in robotic legs, due to its advantages in terms of the mechanical regulation of leg mechanics and energetics and the passive response to abrupt external disturbances during locomotion. This article presents a review of the exploitation of physical compliance in robotic legs. Particular attention has been paid to the segmented, electrically actuated robotic legs, such that a comparable analysis can be provided. The utilization of physical compliance is divided into three main categories, depending on the setting locations and configurations, namely, (1) joint series compliance, (2) joint parallel compliance, and (3) leg distal compliance. With an overview of the representative work related to each category, the corresponding working principles and implementation processes of various physical compliances are explained. After that, we analyze in detail some of the structural characteristics and performance influences of the existing designs, including the realization method, compliance profile, damping design, and quantitative changes in terms of mechanics and energetics. In parallel, the design challenges and possible future works associated with physical compliance in robotic legs are also identified and proposed. This article is expected to provide useful paradigmatic implementations and design guidance for physical compliance for researchers in the construction of novel physically compliant robotic legs.

## 1. Introduction

Legged robots have been among the most active areas in robotic research, which is reflected in the large number of publications on this topic over the last few decades [1,2,3,4,5,6]. One reason for such intense interest is that legged systems could provide better mobility and agility over unstructured terrains in comparison to the wheeled and tracked designs. Such superiority is largely attributed to the use of isolated footholds instead of continuous ground contact, which has been unequivocally demonstrated by animals on foot. Meanwhile, legged robots can be also used as physical platforms to investigate and disclose underlying biological principles, for example, in the biomechanics and neuroscience fields [7,8]. With these robotic platforms, it becomes possible to perform repeatable and parameterized hypothesis testing that is otherwise not biologically possible.

It has proven to be technically challenging to construct legged machines that are agile, energy-efficient, and robust. Legged locomotion is a complex interaction between a large number of joints (e.g., 12 joints for a quadruped, 18 for a hexapod), which requires corresponding mechanical structures, driving elements, and electronics to power and coordinate the robot, making the robot heavy and bulky. Furthermore, due to the unique way of moving, legged robots have to alternately switch their legs between distinct swing and stance phases, resulting in continuous accelerations and decelerations of the robot parts, thereby increasing energy usage and actuation burden. In addition, the discrete moving patterns also cause the leg to repeatedly collide with the ground, and, as a result, the leg has to entail relatively high impact loads. Consequently, these factors impose severe constraints and tradeoffs between the powerful actuation, high strength, light weight, low inertia, and compactness of robotic legs.

When we look at nature, on the other hand, legged animals seem not to be trapped in these tricky issues. It is becoming increasingly clear in recent years that the compliant properties embedded in the musculoskeletal structures of animal limbs play a significant role [9,10,11,12,13,14]. These compliant elements are able to regulate the mechanical and energetic characteristics of muscles and provide passive impact attenuation and adaptivity. During legged locomotion, such contributions of compliant properties could provide help at least in three aspects [15]: (1) Regulating muscle power, where compliant mechanisms can temporarily reinforce the power output of muscles if they release energy more rapidly than it is stored [16]. Typical examples of power reinforcement are animals performing jumping motions, such as locusts and frogs [17], where they usually preload slowly and accelerate their limbs rapidly. In addition, compliant elements can also act as protective mechanisms against damage to muscles, via reducing peak power input to muscle contractile elements. (2) Conserving and recycling energy: It has been found that a sizable fraction of energy in a step can be provided by the compliant tissues, such as tendons and ligaments, rather than by muscle work. For instance, tendons and ligaments in the feet of wallabies have been estimated to store and return 33% of the kinetic and potential energy lost as strain energy [18]. (3) Moderating foot-ground impact forces at touchdown: The compliant elements are able to provide with instantaneous buffering function almost at the very beginning of the impact occurance [19]. One such example is in the human Achilles tendon, the biggest tendon in human body, which significantly contributes to protecting humans from being hurt when jumping and running [20].

With these ideas in mind, roboticists have been investigating the exploitation of compliant elements in legged robot designs. Raibert is widely regarded as one of the pioneers in this field: He and his colleagues have constructed a one-legged hopping robot including compliant elements (air springs) in the leg design since the early 1980s [1,21]. Since that time, a range of engineering prototypes, integrating various forms of compliant elements, have been constructed and reported, ranging from monopedal and bipedal to quadrupedal and hexapodal robots. These robots make use of either mechanical springs or specialized compliant mechanisms in different locations of their legs to achieve the desired compliance behaviors. Recently, several studies have also looked at the integration of compliant spine mechanisms in legged robots, for example, to increase locomotion speed and energy efficiency [22,23,24]. These investigations increase the performance of legged robots and meanwhile advance our understanding towards biological compliant mechanisms.

The motivation of this article is to provide an overview of the exploitation of physical compliance in robotic legs. Several terms have been used in literature to refer to the same concept, i.e., elasticity, flexibility, and compliance. Throughout this article, the term physical compliance is adopted, because it straightforwardly indicates that the compliant behavior is derived from the intrinsic property of physical structures themselves, rather than through active feedback and control. Also, there are different morphologies of robotic legs that have been developed, however, our focus here is specifically on segmented ones. This is because almost all the legged animals have multi-segmented legs, and segmented designs are conducive in terms of, for example, behavioral diversity and self-stabilization [25,26,27]. As a result, robots with telescopic and curved leg designs (for example, RHex-like robots [28,29,30]) will not be covered in this review. In addition, while there have been some robotic legs adopting different actuator principles, such as hydraulic or pneumatic actuators [31], they are not covered here either, because the topic of interest is electromechanical implementation as far as this article is concerned [32].

**Figure 1:**
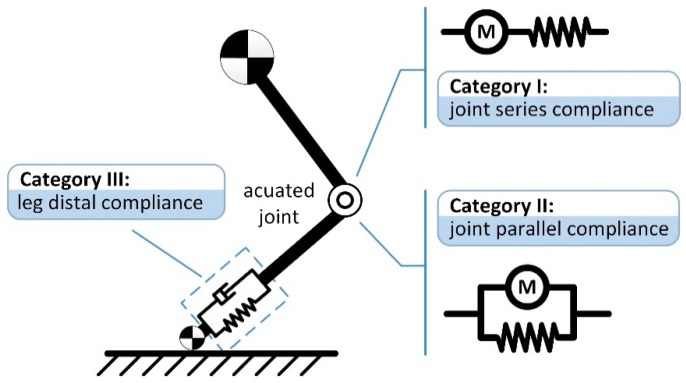
Schematic representation of physical compliance categories in a typical segmented, electrically actuated robotic leg. The utilization of physical compliance is divided into three categories depending on the setting locations and configurations, i.e., joint series compliance, joint parallel compliance, and leg distal compliance.

Below, we review the utilization of physical compliance in robotic legs by categorizing previous studies into three categories depending on their locations and configurations, i.e., joint series compliance, joint parallel compliance, and leg distal compliance, as illustrated in Figure 1. Joint series and parallel compliance are two typical configurations in robotic joints. Leg distal compliance is also a sort of series configuration but is placed in the leg end instead of joints, for the refinement of leg-ground interaction. In addition, it is possible to integrate multiple categories of compliance in a robotic leg, and this will also be discussed in this review. With an overview of the representative work related to each category, the corresponding working principles and implementation processes of various physical compliance are explained. Subsequently, we analyze in detail some of the structural characteristics and performance influences of the existing designs, including the realization method, compliance profile, damping design, quantitative changes in terms of mechanics, and energetics. In parallel, the design challenges and possible future works associated with physical compliance in robotic legs are also identified and proposed. The review is expected to develop a taxonomy to which a significant number of published compliant legged robots can be mapped, and aid researchers to clarify the performance characteristics and design considerations of different categories of physical compliance.

## 2. Category I: Joint Series Compliance

The utilization of physical series compliance has been extensively explored in robotic applications. A representative paradigm is the series elastic actuator (SEA), which consists of a stiff actuator (e.g., an electric motor) in series with an compliant element (e.g., a mechanical spring) [33]. Studies have indicated that SEAs can provide multiple benefits when compared to traditional stiff actuators, including having lower impedance, higher force-fidelity, and better bandwidth [34,35,36]. Having realized such benefits, the concept of SEA has been integrated to form joint series compliance in the design of robotic legs, as summarized in Section 6.

**Figure 2:**
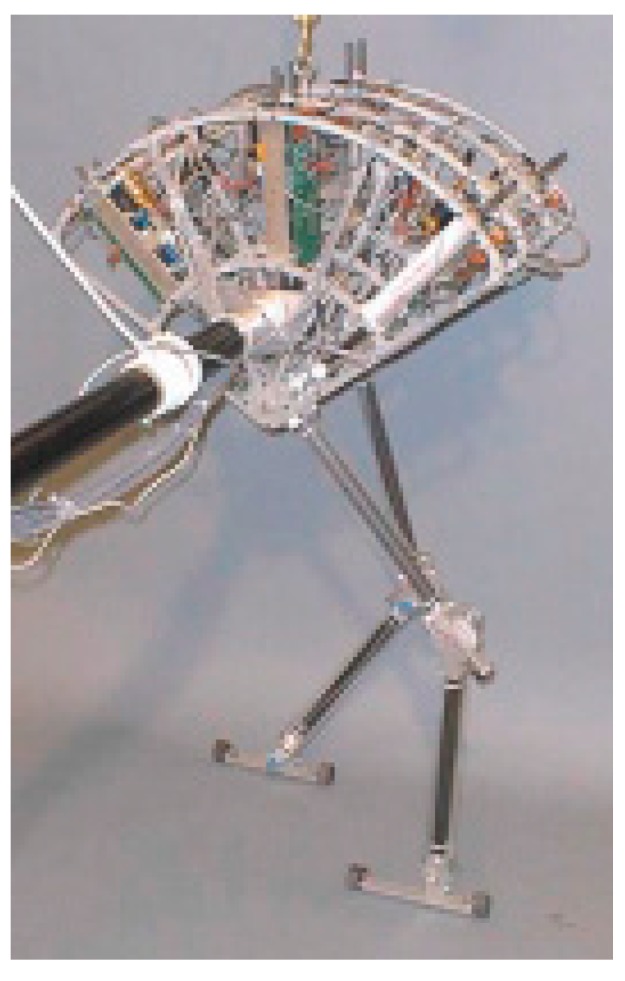
The bipedal walking robot Spring Flamingo.

Spring Flamingo, as depicted in Figure 2, is a planar bipedal walking robot that was built by Jerry Pratt in 1996 [37,38]. The robot has an actuated hip, knee, and ankle on each leg. It is one of the earliest legged robots with joint series compliance implemented by SEAs. Each SEA consists of an electric motor, a timing belt reductor, a ball screw, and four compression springs placed between the transmission system and the output. The four springs therefore form a linear series compliance. In order to reduce leg weight and inertia, the SEAs are located in the upper body, and cable drives are employed to transmit power from the actuators to joints. It is stated that the employment of SEAs allows for the accurate application of torques and a high degree of shock tolerance, and, moreover, inherent robustness and natural dynamics can be exploited in the control of the robot by taking advantage of SEAs. For example, with simple control strategies (e.g., the virtual toe point constraint and the simple force distribution scheme), the robot can walk at a range of speeds and traverse over 15° slopes and rolling terrain. By exploiting the natural dynamics of a kneecap, compliant ankle, and passive swing-leg, the control of the robot becomes easier to achieve and the resultant motion is more natural and efficient. Experiments have shown that the robot can walk as fast as 1.25 m/s and achieve a force control bandwidth of approximately 15 Hz.

**Figure 3:**
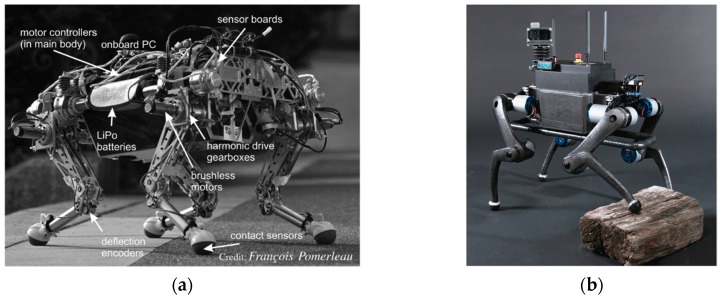
Two quadruped robots with joint series compliance. (**a**) The quadruped robot Starl*ETH*; (**b**) The quadruped robot Anymal, a successor of Starl*ETH*.

Starl*ETH* is a compliant quadrupedal robot developed for versatile, fast, efficient, and robust locomotion, as depicted in Figure 3a. The robot is about 710 mm long, 640 mm wide, 580 mm high, and has a total weight of 25 kg [39]. Each robotic leg has three degrees of freedom (DOF), hip abduction/adduction, hip flexion/extension, and knee flexion/extension. All of the degrees of freedom are equipped with physical series compliance via SEA-based actuation designs. Specifically, for the two degrees of freedom in the hip joint, the series compliance is implemented by two antagonistically pre-compressed springs in combination with a cable pulley (abduction/adduction) and chain drive (flexion/extension), respectively. For the knee flexion/extension, a chain drive as well as a steel cable pulley is employed to connect the knee motor to a compression spring in the shank, thereby forming series compliance. To reduce the leg inertia, all motors and gearboxes are concentrated at the main body. The utilization of joint series compliance makes the robot fully torque controllable, improves its energetic efficiency by intermittently storing energy, and provides necessary robustness for dynamic maneuvers. Hopping experiments with a single leg showed that the robot leg achieves 64% energy recovery and peak power amplification of more than four times. Quadruped experiments have demonstrated that Starl*ETH* has the ability to execute different gaits, ranging from static walking to dynamic trotting gaits, even under substantial external disturbances, such as an unanticipated ground change or external pushes. In particular, building upon the high efficiency and energy preserving mechanisms in joint series compliance, the robot is capable of autonomous long distance (>1 km) trotting with a power autonomy of more than 1 h [39,40,41,42].

The Anymal robot is a direct successor of Starl*ETH*, specifically developed for long endurance autonomous operation in harsh environments, as depicted in Figure 3b [43,44]. Compared to its predecessor Starl*ETH*, Anymal adopts a modular design to simplify system setup and maintenance. It has a full range of joint rotation that allows a higher mobility. This mostly benefits from the modular, highly-integrated, compliant joint unit ANYdrive. The setup of ANYdrive consists of a high-torque motor, a lightweight harmonic drive gearbox in series with a rotational spring, integrated encoders, and electronics, making the robot simple to assemble, handle, and maintain. Using appropriate perception, motion planning, and control strategies, the robot can successfully be used in real-world applications, such as inspection and payload delivery [45,46].

**Figure 4:**
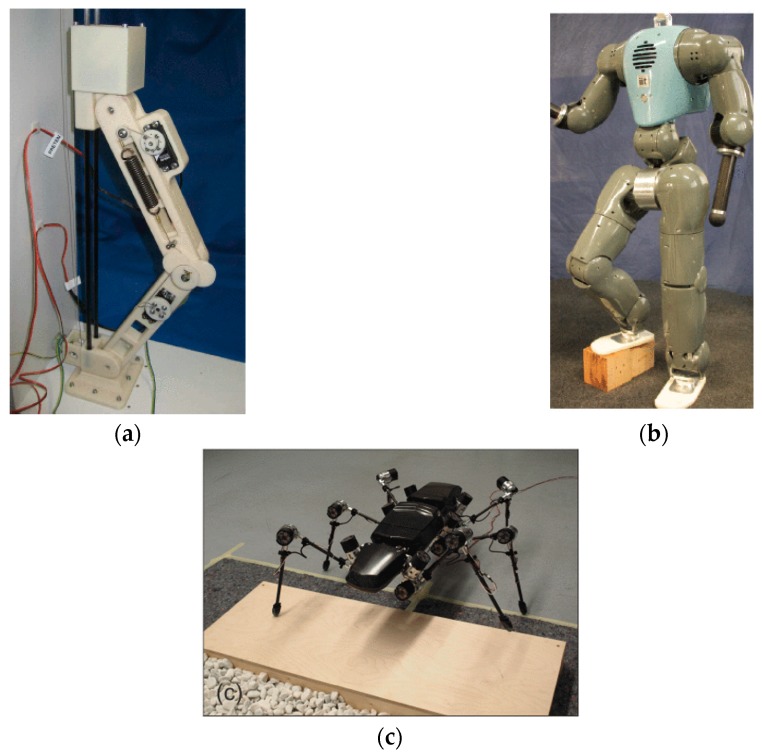
Robots that have self-designed joint series compliance in their legs: (**a**) The single-legged robot Chobino1D; (**b**) The humanoid robot COMAN; (**c**) The hexapod robot HECTOR.

Recently, researchers have investigated the utilization of the so-called nonlinear series compliance or variable stiffness in the joints of legged robots. As shown in Figure 4a, Chobino1D is a one-legged robot whose hip and ankle joints are connected to a vertical slider by linear bearings to implement in-place hopping [47,48]. The knee joint of the robot is powered by an improved design of the compliant actuator MACCEPA [49]. Specifically, the actuation consists of two servomotors, a self-designed heart-shape disk, and a linear extension spring with cable attached. Motor 1 is responsible for controlling the disk, so as to tune the equilibrium position of the actuator. Motor 2 is employed to provide the drive force required to implement hopping motions. The force produced by motor 2 is transferred through the spring and the disk, and eventually arises as the drive torque of the knee joint. With this design, a stiffening nonlinear relationship between the applied torque and the angular displacement of the knee joint is achieved. It has been experimentally demonstrated that the compliant mechanism is able to restore part of the locomotive energy and help reduce the peak power of the motor. Also, it is shown that the achieved hopping height is much higher in comparison to those obtained when a stiff actuator is used.

COMAN is a humanoid robot, roughly 945 mm high and 34 kg in weight, as shown in Figure 4b. The robot totally has a total of 33 degrees of freedom in the legs, arms, body and neck, and 14 of these 33 DOF are equipped with nonlinear series compliance which is achieved by the compliant actuation unit CompACT^TM^ [50]. The setup of CompACT^TM^ consists of a brushless DC motor combined with a harmonic gearbox drive, position and torque encoders, actuation electronics, and a rotary compliant module, including six linear springs. It has been derived that the compliant module can produce a decreasing stiffness characteristic. In addition, a methodology of selecting an appropriate stiffness for the joint spring has been introduced, based on modal analysis and energy storage maximization criteria [51,52].

As depicted in Figure 4b, HECTOR is a six-legged robot designed by following the morphological details of the stick insect *Carausius morosus*. The robot consists of a three-segment body, with each body segment carrying two three-joint legs. The body and leg lengths were scaled-up by a factor of approximately 20, resulting in an overall length of the robot of about 950 mm, leg lengths of about 572 mm, and an overall weight of 13 kg. Similar to Anymal and COMAN, the HECTOR robot is also powered by modular, highly-integrated, compliant joint units. The nonlinear joint series compliance is also achieved entirely by the self-designed sensorized elastomer couplings embedded in the modular units, which is also the distinctive functional feature of the HECTOR robot. It has been claimed that the introduction of nonlinear series compliance not only allows passive adaptation and energy storage, but also enables the application of bioinspired control approaches requiring joint compliance [53,54,55].

## 3. Category II: Joint Parallel Compliance

Aside from series-configured physical compliance in robotic joints, it is also a possibility to place a compliant element in parallel to the motor, thus forming physical parallel compliance. Joint parallel compliance can be viewed from the standpoint of biologically inspired design, mimicking the compliant property within muscles and ligaments, as well as joint capsules. Theoretical studies have revealed that the addition of physical parallel compliance is beneficial to reduce peaks of motor torque and power, and even energetic cost, provided that the parallel compliant mechanism is designed and tuned properly [56,57,58,59,60,61].

**Figure 5:**
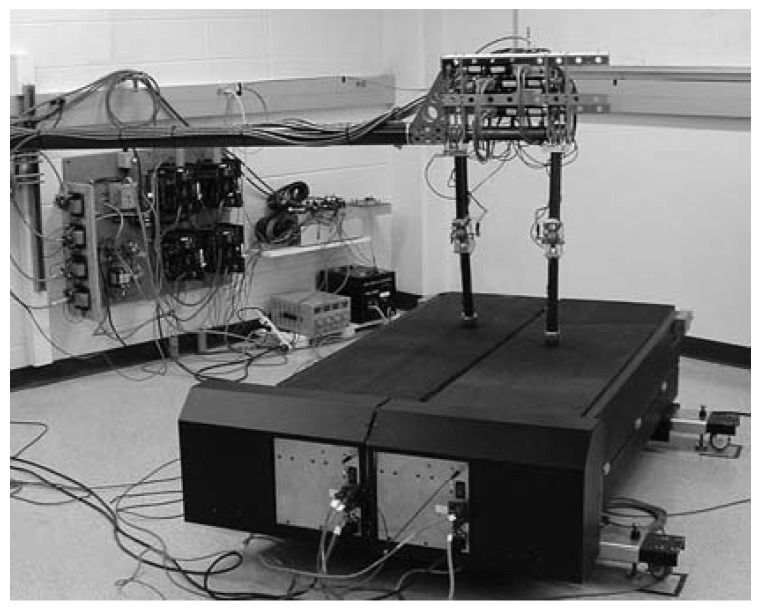
The planar bipedal robot ERNIE, with joint parallel compliance realized by extension springs.

The ERNIE robot, pictured in Figure 5, is a typical planar five-link and electric motor-powered bipedal robot testbed, designed for studying the utilization of parallel compliance. The robot is able to implement planar walking on a treadmill using a hybrid zero dynamics controller. A key design feature of ERNIE is that two linear extension springs are mounted across each knee joint, thereby forming parallel compliance of the knee joint. The authors have stated that the addition of parallel compliance does not increase the control design complexity of the robot. By numerically and experimentally examining the effect of four compliance configurations in the knee joint (one without springs and three with springs of different stiffnesses and preloads), the researchers found that adding springs in parallel with the knee actuators can improve the energetic efficiency of walking, with higher stiffnesses providing greater benefit at higher speeds and lower stiffnesses providing benefit at lower speeds [62,63,64].

**Figure 6:**
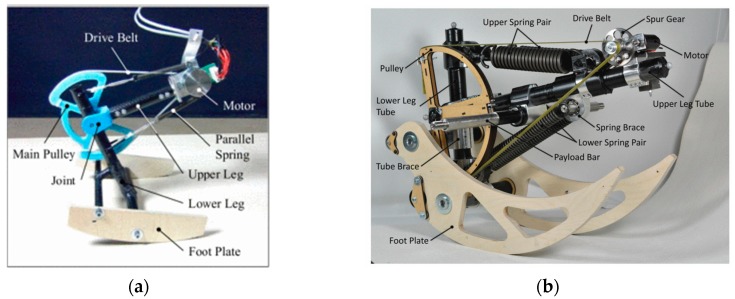
Two curved-foot monopod hopping robots with joint parallel compliance. (**a**) The prototype of CHIARO; (**b**) the prototype of ETH Cargo.

CHIARO and ETH Cargo (shown in Figure 6) are two curved-foot monopod hopping robots with a similar structure and morphology, developed by Iida et al. [65,66,67,68]. The legs of each robot include two segments connected with a rotational joint. In contrast to most monopedal robots, where external supports such as booms or slides are necessary, CHIARO and ETH Cargo adopt a two-plate foot design to prevent the robot from falling laterally, thereby achieving a save stand. The rotational joint of CHIARO is actuated by a motor and transmitted with a drive belt, while ETH Cargo is powered by a motor together with drive belt and a spur gear two-stage reduction. It is worthy of note that the joints of both robots are equipped with physical parallel compliance implemented by linear extension springs. Benefiting from the parallel compliance design, the two robots are able to achieve stable and efficient forward hopping over a wide range of parameters and forward-speeds, with only feed-forward torque control. Particularly, the ETH Cargo robot is able to carry payloads of at least 3 times its bodyweight and achieves a total cost of transport of 0.10, which outperforms the most efficient legged robot so far, additionally keeping up with the most efficient legged animals.

**Figure 7:**
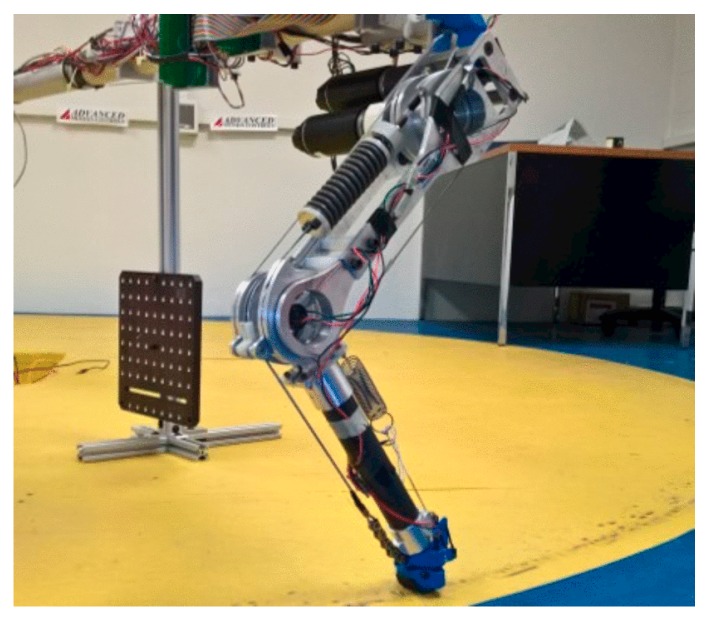
The two-segmented monopedal robot SPEAR with switchable joint parallel compliance. Switchable parallel compliance is employed in its knee joint.

The primary difficulty with the employment of joint parallel compliance is how to improve its downside during the swing phase. To address this challenge, various design measures, for example adding clutch mechanisms, have been taken [69]. As shown in Figure 7, SPEAR is a two-segmented (thigh and shank) monopedal robot, whose torso is attached to a horizontal boom and whose toe is connected to the shank via a passive prismatic joint [70,71,72]. The robot is actuated by two brushless motors placed in proximity to the torso. One is responsible for the hip joint and the other is responsible for the knee joint, operated through a cable-pulley transmission. A key design feature with SPEAR is the ingenious integration of the switchable parallel compliance in the knee joint. Specifically, an energy-storing mechanical spring was added, which wraps around the knee joint, with one end connected to the thigh and the other end attached to a roller chain. The roller chain passes through the toe, where its other end is attached to the shank via a soft spring (see Figure 7). In this design, the toe plays a crucial role as a mechanical switch, subject to the toe-ground contact force, engaging the energy-storing parallel spring during stance and disabling it by connecting the two springs in series during flight. Experimental results demonstrate that 64% of the positive mechanical energy can be provided by the parallel compliance in one stride.

**Figure 8:**
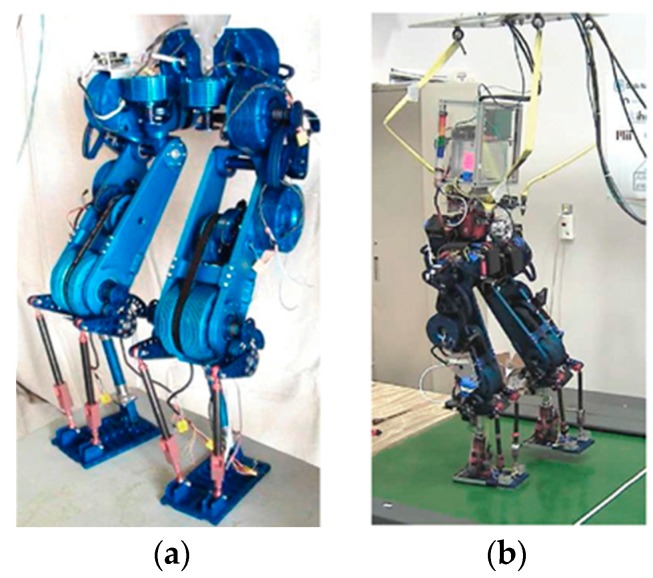
Two curved-foot monopod hopping robots with joint parallel compliance. (**a**) The prototype of CHIARO. (**b**) The prototype of ETH Cargo.

STEPPR (Figure 8) is a bipedal robot that has been designed to explore efficient bipedal walking at Sandia National Laboratories [73,74]. The robot has six degrees of freedom in each leg and uses powerful frameless motors in combination with low-reduction rope transmissions and high-fidelity torque control to achieve highly backdrivable and efficient motions. With a collection of joint motion data analysis and bench level validation, unique parallel compliant mechanisms were designed and incorporated into the hip adductors and ankle flexors of the STEPPR robot. Walking data with the STEPPR robot have shown that the addition of parallel compliant mechanisms at the hip and ankle joints significantly reduce the required actuator energy at those joints, across a variety of gaits and speeds.

## 4. Category III: Leg Distal Compliance

It is also commonly adopted to integrate physical compliance in the distal segment of a robotic leg, which is named leg distal compliance. Because the distal segment of a leg is usually the first part that directly interacts with ground, one of the main functions of leg distal compliance is to passively buffer the foot-ground impact at touchdown, just as the Achilles tendon (the largest and strongest tendon in human body, located at the end of human leg [75,76]) does during human locomotion. Compared to active regulation of the foot force via control schemes, for example, impedance control, a prominent feature of this approach is its rapid response, owing to the mechanical feedback loop formed by the physical compliance, which is particularly crucial to cope with the initial foot-ground force. Additionally, using the compliant segment instead of conventional mechanical links could decrease the rotational inertia of the leg, which is advantageous for minimizing energy consumption and impact impulse [77].

**Figure 9:**
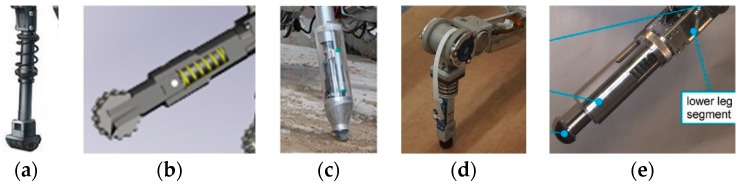
Representative robotic legs that have distal compliance designed based on linear springs: (**a**) BigDog quadruped robot; (**b**) LittleDog quadruped robot; (**c**) Lauron V hexapod robot; (**d**) HITCR-II hexapod robot; (**e**) HyQ quadruped robot.

Perhaps the most direct way to implement distal compliance is to place a mechanical spring in series with the foot in each of their legs. Notable recent examples include the world-famous quadruped robots Bigdog [78] and Littledog [79], designed by Boston Dynamics, depicted in Figure 9a,b. Unfortunately, there are few scientific reports that exist for the design specifications of such distal compliance. Other robots adopting a similar design include the hexapod robots LAURON [80] and HITCR-II [81,82], depicted in Figure 9c,d. With only a simple mechanical design, the distal compliance thus formed can play a crucial role in terms of ensuring safe foot-ground contact. Another robot worth noting is the quadruped platform HyQ, despite not being electrically actuated (Figure 9e) [83]. The researchers that made the HyQ robot investigated the effect of linear springs with different stiffnesses for reducing impact force after landing, finding that foot-ground impact can be reduced significantly in this case, even by more than 60% [83,84].

**Figure 10:**
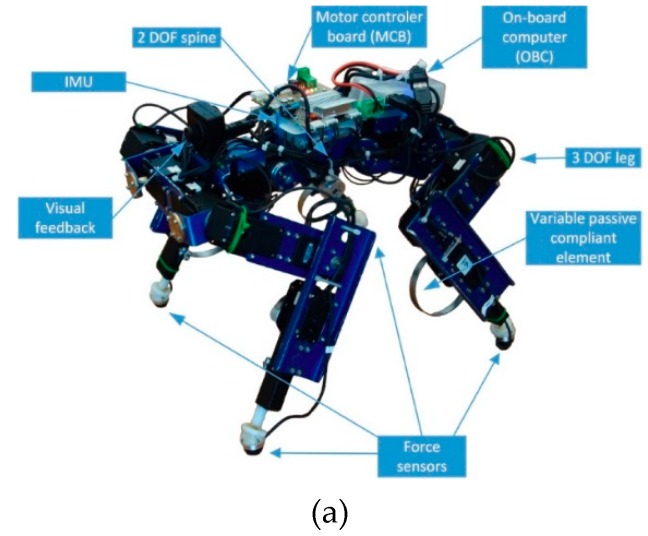

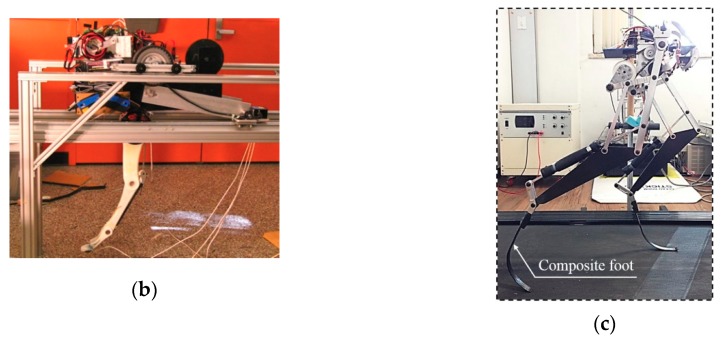
Robots that have self-designed distal compliance in their legs: (**a**) A small-scale quadruped robot. A variable compliant mechanism is designed in the distal segment of its legs; (**b**) The leg of the Massachusetts Institute of Technology (MIT) robotic cheetah; (**c**) The bipedal robot Raptor, with a compliant foot structure made of carbon/epoxy composite material.

Several researchers have also considered the employment of nonlinear leg distal compliance. As a representative example, Figure 10a displays a small-scale electrically driven quadruped robot which has four identical three-segment legs [85,86]. Each leg joint of the robot is powered by a DC servo motor in connection with high-ratio gear reduction. Joint position and foot force sensors are equipped to implement local feedback control in each joint and each leg. The particular novelty of this robot is the integration of a custom designed compliant mechanism as a lower leg implant, which offers nonlinear leg distal compliance for the robot leg. The compliant mechanism consists of a spiral-shaped spring, manufactured from a thin metal sheet, a piston/slider mechanism utilized to transfer the ground contact force to the spiral spring, and a small DC motor that holds and actively rotates the spiral spring. A force sensor is placed at the end of the piston mechanism and serves as a foot to directly contact with the ground. Together with an active compliance control scheme, the robot is able to execute stable locomotion, even in the case of disturbances from unstructured terrain or external pushes. It is claimed that the variable leg distal compliance ensures the filtering of sudden impacts during locomotion.

Figure 10b depicts a prototype of the leg of the Massachusetts Institute of Technology (MIT) robotic cheetah [77,87]. The robotic leg has an electric motor-actuated hip and knee. The novelty of the design lies within the distal ankle joint, in which a compliant design called tendon-bone co-location has been adopted. Specifically, Kevlar webbing is employed in this design, with one end wrapping around the distal ankle joint and the other end attached to a nonlinear spring, realized by a silicone rubber block. By both analysis and a bench level running test, it has been shown that the employment of tendon-bone co-location leads to a stress relaxation on the leg structure by up to 59%, thereby providing for reinforcement of the leg against the ground impact force. In addition, the researchers claim that the introduced distal compliance allows for the storage of impact energy to help improve acceleration during lift-off.

In the realization of leg distal compliance, composite materials are also utilized due to their remarkable mechanical properties. A relevant example is the bipedal robot Raptor, as shown in Figure 10c [88,89]. Each robot leg is composed of an under-actuated 9-bar linkage structure with only one actuated motor, for the sake of being light in weight and having a small moment of inertia. A compliant foot structure made of carbon/epoxy composite material was developed and employed. By performing dynamic running tests with two different foot designs, namely, a rigid aluminum foot and the proposed compliant carbon/epoxy foot, it was reported that the acceleration at the robot body through the composite foot was 40% lower than that through the aluminum foot, indicating the composite foot is able to effectively buffer ground impact during running. Moreover, the robot speed with the compliant carbon/epoxy foot was 23.7% faster than that of the aluminum foot, due to the function of impact energy recycling in the compliant foot.

The HIL leg is a bipedal robot leg [90]. The leg is 1.24 m long (from hip to foot) and weighs 11 kg, where the weight is primarily concentrated at the hip in order to minimize rotational inertia. Both the hip and knee are actuated by motors, together with a harmonic drive or a harmonic drive and belt transmission combination. The novelty of this robotic leg lies within the distal shin that includes a fiberglass leaf spring in parallel with a magneto-rheological damper, for the specific purpose of improving the impact response capability of the robot during dynamic locomotion, such as running and drop jumping. The leaf spring is employed to offer buffering of the ground impact by deformation, while the magneto-rheological damper is used to dissipate impact energy and, as a result, to prevent the leg from bouncing after impact. By implementing an optimal impedance control, the leg design was verified by simulations and experiments using a prototype. It was demonstrated that a safer and more stable landing was achieved in the dropping tests.

## 5. Integrating Multiple Categories of Compliance in A Leg

**Figure 11:**
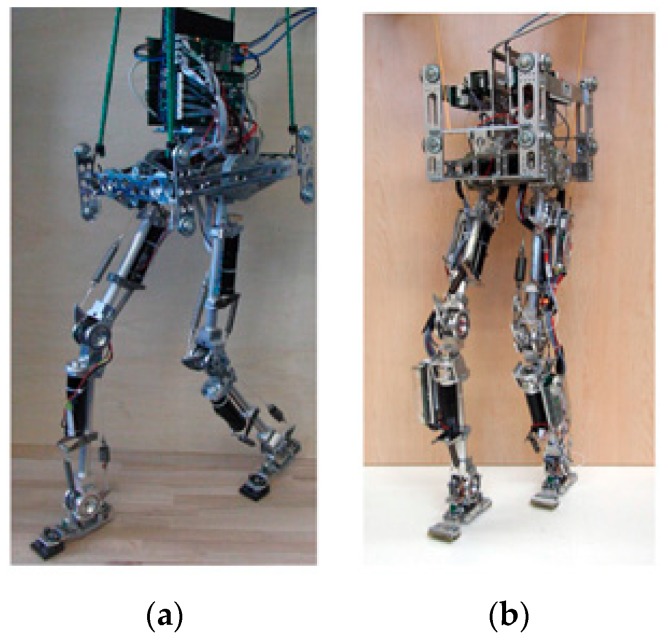
The BioBiped series robots: (**a**) BioBiped1; (**b**) BioBiped2.

Several studies have also looked at the integration of multiple categories of compliance in a robotic leg, for the purpose of taking advantage of different compliance designs. A representative example is the BioBiped series of robots, a series of bipedal platforms designed to investigate human locomotion. Three generations of prototypes, named BioBiped1, BioBiped2, and BioBiped3, have been presented. All three platforms have a similar structure, size, and morphology, consisting of two 3-segmented legs and a small torso. Each leg has two rotational degrees of freedom (pitch and roll) in the hip and one rotational degree of freedom (pitch). As depicted in Figure 11, for BioBiped1 [91] and BioBiped2 [92], the hip joint is powered by a SEA which is realized by a geared electric motor in connection with built-in translational springs. The knee and ankle joints adopt SEAs to achieve extension motion, while the flexion motion is performed passively by a translational spring with cable connections. Besides, three spring-cable units spanning two joints are incorporated into each leg, to mimic the biarticular muscles found in humans. As a result, these passive spring-cable units form the parallel compliance of the corresponding joint actuators. The authors claimed that these passive spring-cable units play an important role in transferring energy and coordinating the synchronization of the leg joints.

**Figure 12:**
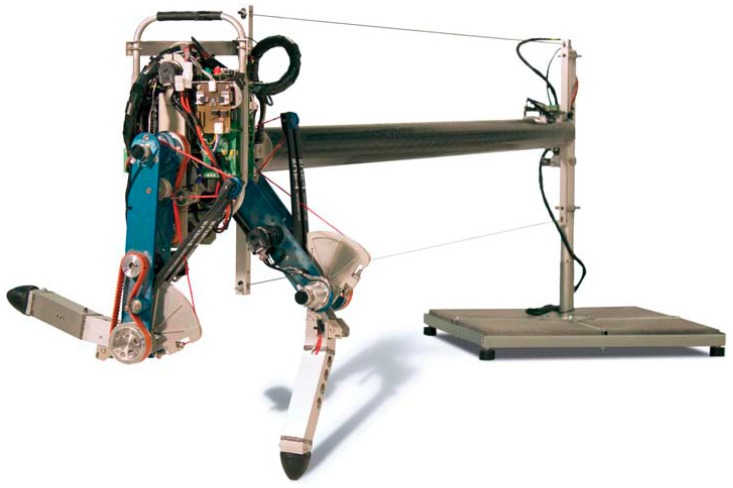
Running robot Phides, with both series and parallel compliance in the knee joints.

Phides is a bipedal running robot, consisting of a torso and two kneed legs [93]. The torso is rigidly connected to a horizontal boom to achieve planar motions. A key design feature with Phides is the knee actuation, which includes both series and parallel compliance, as shown in Figure 12. The series compliance is implemented with a torsion bar and is responsible for eliminating large shocks at touchdown. The parallel compliance is achieved through a fiberglass leaf spring alongside a nonlinear transmission and a latching mechanism. The latching mechanism allows the enablement of parallel compliance for energy storage and release during the stance phase, while detaching it to permit free joint rotation during flight. Using a state-machine based controller, the robot is able to achieve humanlike running. It has been reported that, owing to the integrated physical compliance, the required peak motor power was reduced by 26%, which allows the robot to achieve an unprecedented flight time of 54% of the stride.

**Figure 13:**
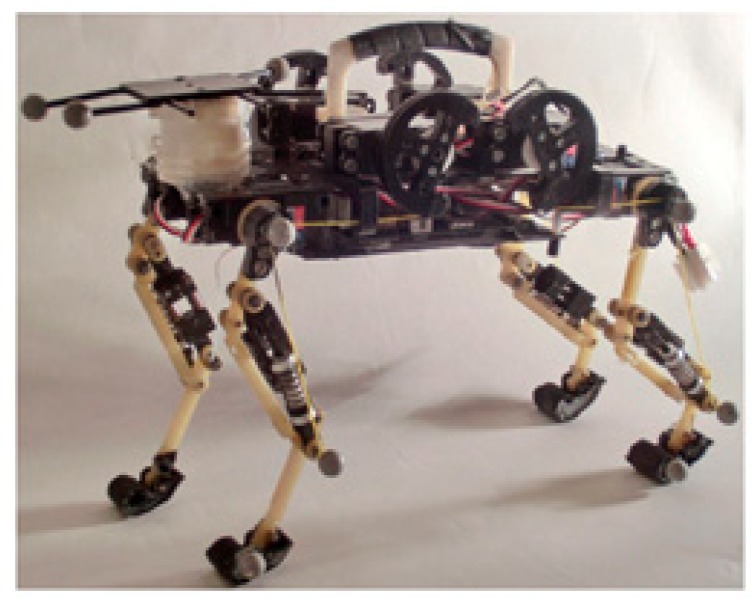
The Cheetah-cub quadruped robot.

Cheetah-cub is a small quadruped robotic platform with compliant and segmented legs [94,95]. As shown in Figure 13, the robot leg is actuated by two servo motors. One responsible for the hip joint and the other for the knee joint, via a cable mechanism. Three springs are employed in this leg design. In the second segment, a diagonal spring is used in parallel to the knee actuation, therefore forming joint parallel compliance. The second is a biarticulated spring element that was implemented as a replacement of a rigid parallel link in the second segment. Under tension, this spring provides series leg compliance. Besides, a helical spring is located in the most distal leg joint, thereby providing leg distal compliance. It was found from hardware experiments that, benefiting from the added compliance elements, the robot shows a self-stabilizing behavior over a large range of speeds with open-loop control and achieves the fastest locomotion among quadruped robots below 30 kg.

## 6. Discussion and Outlook

In this article, we presented an overview of the three main categories of physical compliance currently used in robotic legs. Part of the robots with joint series or parallel compliance in legs are summarized in Table 1. It has been demonstrated that multiple benefits in terms of mechanics and energetics can be obtained when integrating properly compliant elements into joints or leg distal segments. The first category is joint series compliance. Introducing series compliance in a leg joint is advantageous to increase the robustness to external perturbations, improve the energy efficiency, and realize accurate torque control. Joint series compliance is usually of high-stiffness and low-deflection, so as to transmit motor torque. In contrast, the allowable deflection of joint parallel compliance is relatively larger, in order to assist with joint motion. It has shown that the reasonable joint parallel compliance is beneficial to reduce the energy consumption and power requirements. A possible drawback of joint parallel compliance is that it may hinder leg movements during the swing phase. Therefore, an efficient switch mechanism is required to facilitate the employment of joint parallel compliance. For the third category, i.e., leg distal compliance, the primary aim is to buffer high levels of ground impact at the very beginning of the impact occurrence, thereby preventing structural damage of the robot leg. In dynamic locomotion, distal compliance is also helpful to increase the take-off speed by recycling the impact kinetic energy. Apart from these factors, it has also been reported that potentiometers on passive compliant joints are able to provide versatile sensing, for instance, sensing joint position and force/torque [96].

The addition of physical compliance in robotic legs is not an easy job. On one side, the benefits in terms of the mechanical regulation of leg mechanics and energetics and passive response to ground impact can be obtained if integrating properly compliant elements into leg joints or distal segments. On the other side, it may also complicate the mechanical design and thus the subsequent system modelling and control, which is a major downside. Table 2 summarizes the structural features and resultant performance changes of most of the above-mentioned compliant legged robots. The summarized structural features include the realization method, compliance profile, and damping utilization. The performance data, in terms of energy usage, power requirements, and foot-ground impact, were collected and quantified in a relative manner to avoid unfair comparisons (so long as the data were available).

### 6.1. Realization Method

Different realization techniques of physical compliance in robotic legs have been proposed. As shown in Table 2, the realization methods can be divided to three major classes, namely, extension spring-based, compression spring-based, and customized compliant components. Among these designs of physical compliance, using linear mechanical springs is a common practice, primarily because mechanical springs are the most commercially available off the shelf compliant elements and are easy to model and control. For the extension spring-based realization method, cables or steel wires are usually used to attach the springs into the drive chain, and as a result, compliance categories I and II could be achieved. For instance, the electrically actuated robot BioBiped1 integrates multiple extension springs, each attached with cables, to implement series compliance in the hip, knee, and ankle joints (compliance categories I), achieving a roughly 33% and 35% reduction in energy loss and ground impact, respectively [97]. Additionally, with cable transmission, it is possible to reduce the effective inertia of the legs by locating heavy motors on robotic bodies, such as the robot Spring Flamingo does [37]. The robot SPEAR realizes parallel compliance in the knee joint (compliance categories II) using two extension springs (one hard and one soft) attached by steel cables, suggesting that 64% of energy contribution can be provided by the parallel compliance in one stride [71]. To achieve nonlinear compliance, a simple example is the quadruped robot Puppy, which uses a linear spring attached across its knee joint, and, as a result, an effective nonlinear torsional spring is generated as the lever arm changes [98]. Another excellent example worth highlighting is the robot Chobino1D. The robot achieves nonlinear series compliance in the knee joint (compliance categories I) using a linear extension spring and a self-designed profile disk. With this design, the robot achieves a 32% energy reduction and around 60% peak power reduction [47].

Unlike the extension spring-based realization method, in which hardware complexity is not significantly increased, compression springs are usually embedded into the mechanical structures of legs and play a role in the transmission loop. Take the robot COMAN as an example, where a miniature compliant module including six compression springs has specifically been designed and placed into the joint actuation [50]. In addition, it can be seen from Table 2 that the compression spring-based realization method could be applied to perform compliance categories I and III for energy storage, power amplification, and ground impact reduction in robotic legs. Starl*ETH* is a representative example, adopting compression springs to realize joint series compliance (compliance categories I). With the applied compliant design, the robot leg achieves 64% energy recovery and peak power amplification of more than four times in hopping experiments [40,42]. As another example, the HyQ robot employs compression springs to achieve distal compliance (compliance categories III), resulting in as much as 60% of the landing impact being reduced [83,84].

The third realization method is to make use of customized compliant components. This method has been adopted by some robots to realize compliance categories I (e.g., HECTOR), II (e.g., STEPPR) and III (e.g., MIT cheetah leg). By tailoring specific compliant components, it is possible to produce a desired nonlinear compliance profile specialized for particular tasks (e.g., human-like walking with the STEPPR robot). Apart from customizing metal compliant components, an idea worth highlighting is to make use of composite or elastomer materials, which promise to achieve a smaller size, lighter weight, and higher performance. For example, the Raptor robot makes use of a foot structure made of a carbon/epoxy composite material whose mass is 3 times lower than the conventional aluminum foot. Owing to the composite foot structure, the Raptor robot achieves a 40% reduction in the impact load and a 23.7% improvement in speed [89]. The HECTOR robot employs an elastomer coupling in its modular drive unit. Compared to the compliant combination realized with compression springs (roughly 55 mm in diameter and 0.1–0.2 kg in weight), the elastomer coupling is only 20 mm in diameter and ~0.05 kg in weight, despite the different design requirements (values here are estimated from the corresponding publications) [50,54].

### 6.2. Compliance Nonlinearity

Most of the compliant robots designed in early period employ linear compliance behaviors that are implemented with mechanical springs. More recently, researchers have been attempting to investigate the design and effects of various nonlinear compliant behaviors in robotic legs. Table 3 summarizes the specific nonlinearity of physical compliance currently used in several robotic legs. From the table, it can be seen that different types of nonlinearity (e.g., softening, stiffening, and non-monotonic) are adopted in robotic legs, even within the same category of compliance, revealing that, at present, it is still open as to what type of nonlinearity is a good fit.

Interestingly, when we look at nature, a similar nonlinearity of compliance behaviors is always exhibited in biological muscles and tendons. For example, biologists have found, by measuring the passive force-length (compliance) relationships in different muscles of different animals, a stiffening or even exponential tendency is always presented [99,100,101,102]. Likewise, similar nonlinear behaviors have also been experimentally identified in the tendons of different animals [103,104,105]. All these experimental pieces of evidence essentially reveal a universal preference in biological compliance. With these in mind, it is necessary to investigate the underlying mechanisms behind the similarity in biological compliance by, for example, biomechanics research, and, hopefully, to distill a general strategy for the design of nonlinear compliance in robotic legs.

Having discussed the possible benefits from nonlinear compliance, we have to think about their hardware realization in robotic legs. Compared with the design of linear compliance, implementing nonlinear compliance usually requires additional mechatronic design, which could lead to bulky and complex designs. Using elastomer and composite materials may allow for more compact and robust designs by reducing the number of support structure, however, these materials would inevitably introduce other tricky issues, for instance, hysteresis [54]. Thus, from the perspective of hardware realization, one has to think about whether or not the performance enhancement outweighs the complexity increase involved in doing so on the side of the overall system. Finding the optimum point between system complexity and desired performance is key to the success of the utilization of physical compliance in robotic legs.

### 6.3. Versatility and Adaptation of Physical Compliance

Generally, compliance behaviors in a robotic leg can be divided into two different types, namely, active compliance and passive compliance. Active compliance means that the compliance is created by controlling actuators according to feedback measurements, just like having a spring-damper in the leg. A great advantage of this method is its versatility. The compliance characteristics can be flexibly modulated on demand. However, passive compliance is created by integrating compliant elements (like mechanical springs) into the leg structure. A prominent feature of such compliance is its rapid response to impact forces, owing to the mechanical feedback loop formed by the physical spring. However, in contrast to active compliance control, which allows the adjustment of related parameters during the course of operation, physical compliance properties, regardless of linear or nonlinear compliance profiles, have to be set and calibrated during the design and manufacturing process, and cannot be tuned in situ. As a compromise, current physical implementations of compliance behaviors in robotic legs are just chosen for a specific motion mode, for example, slow walking or fast running. Consequently, this inevitably reduces the versatility of physical compliance for other modes of motion, and thus the behavioral improvement of the robot. In addition, even with the same motion mode, the state changes in the robot itself, for example, the loading variation, may also correspond to different degrees of compliance properties.

It has been revealed that the mechanical properties of biological compliance are changeable and can be reshaped through rapid modulation or long-term training [14]. For example, there is increasing evidence that tendons can be rapidly strengthened in response to heavy external loads through increasing blood flow [106,107]. Besides, tendon mechanical properties can be also varied by long-term exercise [108,109]. Such mechanisms improve the versatility and adaptation of passive biological compliance for different locomotion and manipulation tasks. 

Regarding the physical compliance in robotic legs, it is also necessary and promising to improve the versatility and adaptation by enabling the in situ tuning of physical compliance behaviors according to locomotion tasks. In this context, some designs achieve a certain degree of compliance adjustment by adding another motor in the compliant mechanisms [47,85,110]. In addition, Sharbafi et al. have proposed the concept of hybrid electric-pneumatic actuators (EPA), applying them to the legs of the BioBiped3 robot to achieve the effective tuning of compliance [111]. The EPA consists of an electric motor and a pneumatic artificial muscle (PAM) as a replacement of the traditional spring-based compliant mechanisms. Utilizing PAM, the compliance behaviors can be altered across a wide range by tuning the PAM air pressure. To the best of the authors’ knowledge, this is the only work so far that is able to achieve a significant tuning of physical compliance. Besides this, it is also possible to make use of functional materials capable of reversibly changing mechanical properties in response to external stimuli, such as heat and electrical current [112,113]. Using these smart materials is not only beneficial to improve the versatility and adaptation of physical compliance, but also provides a promising way to construct more compact and lightweight robotic legs as well.

### 6.4. Damping

Most of the literature concerning physical compliance in robotic legs focuses on the property of stiffness. In fact, damping is another important passive property that influences the performance of robotic legs [114,115]. Only a few research works have considered the addition of the damping property (see Table 2). One is the Starl*ETH* robotic leg, in which a damper unit is applied in the knee joint. The damper is active only during the flight phase, so as to prevent undesired joint oscillations [40]. The other work is the HyQ robotic leg, in which a magnetorheological (MR) damper was placed in the distal segment of the leg. The primary aim here was to dampen unwanted oscillations at touchdown by dissipating the impact kinetic energy, and thereby to improve the traction between the foot tip and the ground [84,116]. In addition, an efficient activation mechanism for the MR damper may be required to avoid hindering desired leg movements and, meanwhile, minimizing the power consumption of the damper itself [117].

Apart from suppressing oscillations, theoretical research also reveals that appropriate damping is conducive for locomotion stability [118,119,120,121], system robustness with respect to the design and control parameters [118,119,120], perturbation rejection [119,120], and foot-ground interaction [121,122]. It is necessary to further investigate the implementation and effects of physical damping in robotic legs.

## 7. Conclusions

This article has presented an overview of the utilization of physical compliance in robotic legs and proposed a taxonomy to which various scales and configurations of compliant robotic legs can be mapped. In contrast to some existing review work on compliant legged robots, we have paid particular attention to segmented, electric motor-powered robotic legs, such that we can provide a comparable analysis. After presenting the working principles and implementation processes of representative work related to each category, we have highlighted the realization method, compliance profile, versatility and adaptation, and damping utilization in the current designs. In addition, several possible directions to further facilitate the exploitation of physical compliance in robotic legs have been identified and proposed. First, it is necessary to explore the potential functional benefits behind the preferred compliance nonlinearity in biological systems and to distill a general strategy for the profile design of nonlinear physical compliance. The second research direction is to investigate the introduction and use of physical damping in robotic legs. Third, it is promising to make use of functional materials capable of reversibly tuning compliance in response to external stimuli, such as heat and electrical current. Using tunable compliance materials, it is not only possible to improve the versatility and adaptation of physical compliance, but also to provide a promising way of constructing more compact and lightweight robotic legs. We hope to see more researchers developing novel, high-performance compliant robotic legs. Having realized such benefits, the concept of SEA has been integrated to form joint series compliance in the design of robotic legs, as summarized in Table 3.

## Figures and Tables

**Table 1:** Legged robots with joint series or parallel compliance in legs.

Robot	References	Locomotion Ways	Leg Number	DOF Per Leg	Leg Length (mm)	Leg Equivalent Mass^a^ (kg)	Joints with Compliance
Joint Series Compliance							
Spring Flamingo	[37,38]	Walking	2	3	1000	7	Hip, knee, ankle
Starl*ETH*	[39,40,41,42]	Versatile locomotion	4	3	498	5.75	Hip, knee
Anymal	[43,44,45,46]	Versatile locomotion	4	3	500	7.5	Hip, knee
Chobino1D	[47,48]	Hopping	1	2	400	0.7	Knee
COMAN	[50,51,52]	Walking	2	6 (3 joints)	537	17	Hip, knee, ankle
Joint Parallel Compliance							
HECTOR	[53,54,55]	Walking	6	3	610	2.17	Hip, knee, ankle
ERNIE	[62,63,64]	Walking	2	2	720	9.3	Knee
CHIARO	[65,66,67]	Hopping	1	1	360	0.72	Knee
ETH Cargo	[68]	Hopping	1	1	623	28.6	Hip
SPEAR	[70,71,72]	Hopping	1	2	647	8.07	Knee
STEPPR	[73,74]	Walking	2	6 (3 joints)	--	46.5	Hip, ankle

^a^ Leg equivalent mass is the sum of the leg mass and the mass distributed by the torso. DOF: Degrees of freedom.

**Table 2:** Structural features and performance characterization of physical compliance in robotic legs.

Realization Method	Representative Robot	Compliance Category	Compliance Profile	Additional Damping	Performance Characterization (in Percentage Terms)
Extension spring-based	BioBiped1	I	Linear	No	~33% reduction in energy loss ~35% reduction in ground impact
	ERNIE	II	Linear	No	–
	SPEAR	II	Linear	No	64% of energy recovery
	Chobino1D	I	Nonlinear	No	~32% reduction in energy ~60% reduction in peak power
Compression spring-based	Starl*ETH* leg	I	Linear	Yes	64% of energy recovery 400% amplification in peak power
	COMAN	I	Nonlinear	No	–
	HyQ	III	Linear	Yes	60% reduction in ground impact
Customized compliant components	HECTOR	I	Nonlinear	No	–
	STEPPR	II	Linear	No	~60% reduction in energy (hip) ~43% reduction in energy (ankle)
	Phides	I + II	Nonlinear	No	26% reduction in peak power
	Raptor	III	–	No	40% reduction in impact load 23.7% improvement in speed
	MIT cheetah leg	III	–	No	59% reduction in ground impact

~Values are calculated from the corresponding publication data.

**Table 3:** Compliance nonlinearity adopted in robotic legs.

Robot	Compliance Category	Compliance Nonlinearity
COMAN	I	Softening
HECTOR	I	Stiffening
Chobino1D	I	Non-monotonic
